# Convergent Immunopathology in Influenza-Associated Encephalopathy: CD3+ CD4+ T Cell Depletion, IL-6-Mediated Inflammation, and Complement C3 Dysregulation

**DOI:** 10.3390/diagnostics16203290

**Published:** 2026-10-10

**Authors:** Dongmei Zhang, Yang Chen, Ling Hu, Sha Wei, Xiaoxia Lu, Dan Sun

**Affiliations:** 1 Center for Emergency and Critical Care Medicine, Wuhan Children’s Hospital (Wuhan Maternal and Child Healthcare Hospital) Tongji Medical College, Huazhong University of Science and Technology, Wuhan 430015, China; 2 Department of Pediatric Neurology, Wuhan Children’s Hospital (Wuhan Maternal and Child Healthcare Hospital) Tongji Medical College, Huazhong University of Science and Technology, Wuhan 430015, China; 3 Department of Respiratory Medicine, Wuhan Children’s Hospital (Wuhan Maternal and Child Healthcare Hospital) Tongji Medical College, Huazhong University of Science and Technology, Wuhan 430015, China

**Keywords:** influenza A virus, CD3+ CD4+ T-lymphocytes, complement C3, IL-6, innate immunity, cytokine storm, encephalopathy

## Abstract

**Background:** Influenza-associated encephalopathy (IAE) is a severe neurological complication, yet immune biomarkers for its early prediction remain limited. This study aimed to elucidate the systemic immune signature defining IAE by integrating humoral and cellular immunity analyses. **Methods:** We conducted a retrospective cohort study of 623 pediatric influenza patients presenting with fever and convulsions (55 IAE, 568 febrile seizure (FS) group) at Wuhan Children’s Hospital (2020–2025). Serum complement components (C3, C4), lymphocyte subsets (including CD3+ CD4+, CD3+ CD8+, CD19+ B cells, and NK cells), and cytokines (IL-2, IL-4, IL-6, IL-10, TNF-α) were quantified. Multivariable logistic regression was performed, rigorously adjusted for age, sex, influenza subtype, time from fever onset to sampling, prior use of intravenous immunoglobulin (IVIG) or glucocorticoids, and pre-diagnostic convulsive episodes. Sensitivity analysis via 5th-percentile Winsorization addressed floor effects in C3 measurements. **Results:** IAE was associated with distinct alterations in both humoral and cellular immunity. Patients with IAE had significantly lower serum C3 levels and higher IL-6 concentrations compared to controls. Multivariable regression revealed that IL-6 (adjusted OR = 1.002, 95% CI: 1.000–1.003, *p* = 0.037) and CD3+ CD4+ T cells (adjusted OR = 0.983, 95% CI: 0.979–0.988, *p* < 0.001) were independent predictors in the fully adjusted model. Lower serum C3 levels were associated with IAE across sequentially adjusted models (fully adjusted OR = 0.05, 95% CI: <0.001–0.138, *p* = 0.002). Sensitivity analysis using 5th-percentile Winsorization of C3 yielded a consistent direction of association (adjusted OR = 0.019, 95% CI: 0.002–0.226), supporting the robustness of the primary finding. **Conclusions:** These findings identify a convergent immunopathology involving CD3+ CD4+ T cell depletion, IL-6-dominated inflammation, and lower C3 levels in pediatric IAE. Monitoring this immune triad may facilitate early risk stratification. The causal relationships and therapeutic implications of these observations require validation in prospective and mechanistic studies.

## 1. Introduction

Influenza A virus (IAV) remains a ubiquitous respiratory pathogen posing a substantial threat to global public health, particularly among pediatric populations. A subset of children rapidly progress to influenza-associated encephalopathy (IAE), among which influenza-associated acute necrotizing encephalopathy (IA-ANE) is the most severe subtype, characterized by rapid progression and high mortality [1,2]. Clinically, IAE manifests as acute impairment of consciousness, recurrent seizures, and focal neurological deficits, often resulting in severe long-term sequelae in survivors [2].

Crucially, the pathogenesis of IAE is distinct from classical viral encephalitis; it is predominantly a host-driven pathology rather than a consequence of direct viral invasion of the central nervous system (CNS) [3]. During acute IAV infection, hyperactivation of the immune system triggers a systemic “cytokine storm.” Emerging evidence highlights the role of nuclear pore proteins, such as RANBP2/NUP358, in regulating antiviral signaling and hyperinflammation [4]. Concurrently, the complement cascade—a cornerstone of the humoral innate immune system—is increasingly implicated. Experimental models demonstrate that complement C3 plays a complex role during IAV infection; while physiological levels are protective, excessive consumption may exacerbate systemic inflammation and compromise vascular integrity [5]. Furthermore, C1q modulates viral entry and inflammatory responses [6]. However, whether C3 consumption serves as a critical determinant for blood–brain barrier (BBB) disruption in pediatric IAE remains undefined.

Beyond humoral immunity, accumulating evidence implicates profound perturbations in cellular immunity in IAE pathogenesis. In H1N1-associated ANE, pediatric patients exhibit a striking reduction in peripheral CD4+ T cell counts accompanied by heightened expression of exhaustion markers (Eomes, CD69, PD-1) and concordant surges in IL-6 and IL-8 within both cerebrospinal fluid and plasma, suggesting that an activated yet dysfunctional CD4+ T cell compartment may orchestrate severe neuroinflammation [7]. Consistent with this, retrospective analyses of pediatric IAE cohorts have identified diminished CD4+ T cell percentages and reduced CD4+/CD8+ ratios as independent predictors of mortality, with thresholds of CD4+ T < 31.1% or CD4+ T/CD8+ T < 1.33 conferring elevated risk of death [8]. Earlier work further documented upregulated CTLA-4 expression on CD4+ T cells in children with IAV encephalopathy relative to those without neurological complications [9]. Mechanistically, CD4+ T cells are indispensable for coordinating protective anti-influenza immunity—providing helper signals for B cell antibody production, licensing CD8+ T cell cytotoxicity, and sustaining inflammatory resolution [10]; their depletion or functional paralysis may therefore cripple viral clearance while failing to restrain the cytokine storm.

Prior immunophenotyping studies of IAE are limited by small sample sizes and narrow analytical panels, typically focusing on isolated cytokines without integrating the complement–lymphocyte axis [8,11]. Although recent studies have profiled risk factors in Chinese pediatric cohorts [2,12], the specific immunological signature—particularly the interplay between complement consumption, CD4+ T cell dysfunction, and cytokine dysregulation—remains uncharacterized. Here, leveraging a large retrospective cohort of 623 pediatric influenza patients presenting with febrile seizures (55 IAE, 568 FS) at Wuhan Children’s Hospital (2020–2025), we performed comprehensive quantitative profiling of complements (C3, C4), lymphocyte subsets (including CD3+ CD4+ T cells, CD3+ CD8+ T cells, CD19+ B cells, and NK cells), and cytokines (IL-6, IL-10, TNF-α). Our analysis reveals that profound CD3+ CD4+ T cell depletion, coupled with elevated IL-6 and aberrant C3 dynamics, defines a high-risk immunological phenotype for IAE. These findings suggest a “two-hit” model of innate-adaptive immune dysregulation as a hypothesis-generating framework, and identify potential biomarkers for early risk stratification. The causal relationships and therapeutic implications require further validation.

## 2. Materials and Methods

### 2.1. Research Subjects

**Figure 1:**
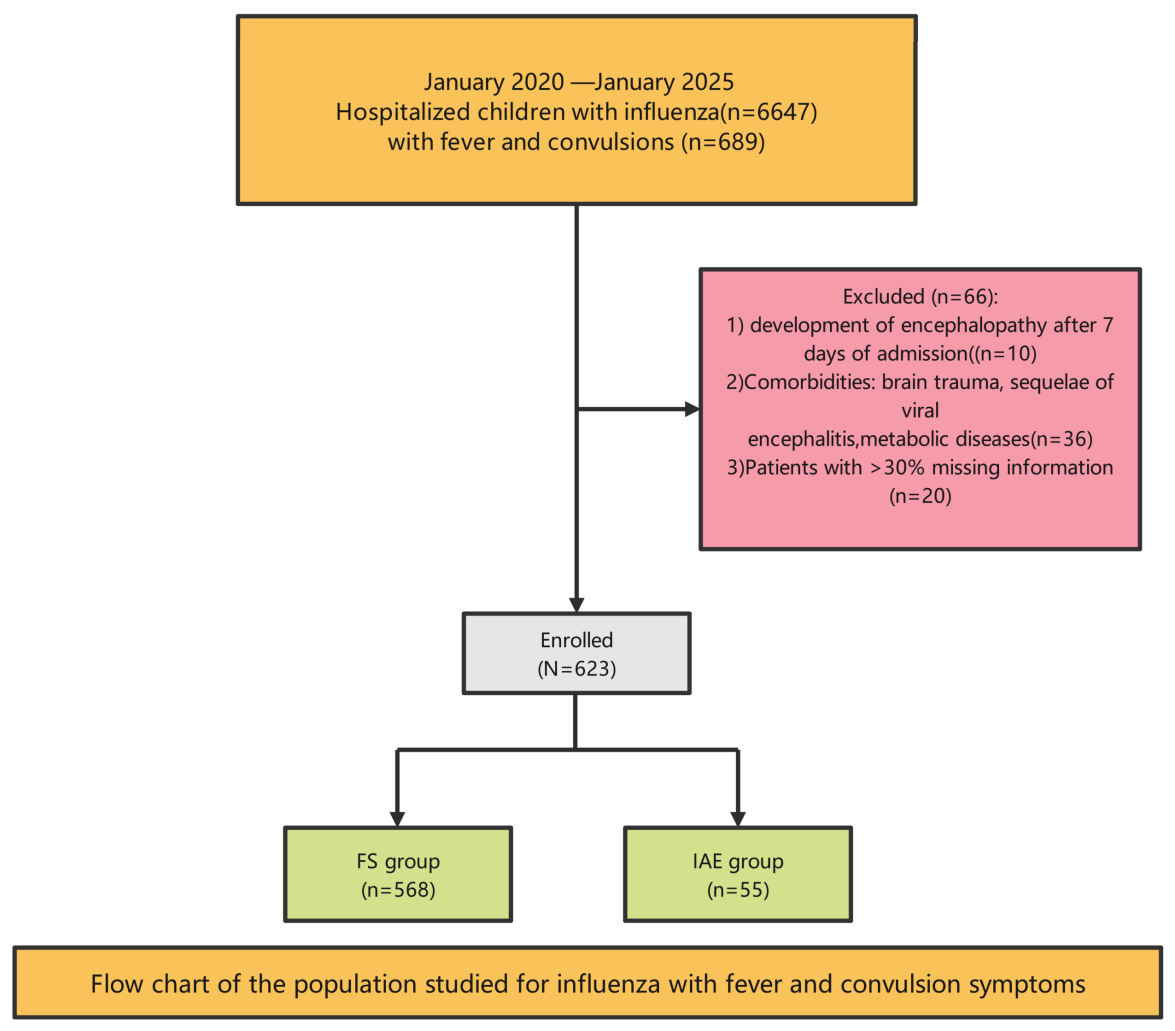
Flow chart of the population studied for influenza with fever and convulsion symptoms. Abbreviations: FS: Febrile Seizures; IAE: Influenza-Associated Encephalopathy.

This study is a retrospective cohort study. Data on 6647 pediatric influenza patients treated at Wuhan Children’s Hospital Affiliated with Tongji Medical College of Huazhong University of Science and Technology from January 2020 to January 2025 were collected. We enrolled 623 influenza patients presenting with fever and convulsions (Figure 1), all of whom had no evidence of co-infections and received oseltamivir after diagnosis, with Nonsteroidal Anti-inflammatory Drugs (NSAIDs) administered for fever management during the febrile phase. This study complied with the standards of medical ethics, and the current study followed the principles of the Declaration of Helsinki, which was approved by the Medical Ethics Committee of Wuhan Children’s Hospital (Approval No. 2024R044-E01) without the need for written informed consent.

**Inclusion criteria:** ① oropharyngeal swab pathogen detection indicating type A (type B) antigen (RNA) positivity and ② clinical symptoms including both fever and seizures. Exclusion criteria: ① development of encephalopathy/neurological complications after 7 days of admission (to focus only on acute course of IAE); ② presence of underlying diseases: brain trauma, post-viral encephalitis, metabolic disorders; and ③ patients with missing information >30%.

**The diagnostic criteria for IAE:**

**Clinical manifestations:** Presence of central nervous system dysfunction (consciousness disturbance with Glasgow Coma Scale (GCS) < 12, seizures, or other neurological deficits) persisting for >24 h.

**Imaging findings:** Head CT or MRI showing cerebral edema, symmetric lesions in bilateral deep gray matter (e.g., basal ganglia, thalamus), or multifocal hemorrhagic necrosis (in acute necrotizing encephalopathy).

**Epidemiological link:** Illness occurring during the influenza season or within 7 days of confirmed/suspected influenza infection.

**Exclusion of other causes:** Ruling out other CNS infections, autoimmune encephalitis, or metabolic encephalopathy.

### 2.2. Research Methods

Clinical data included baseline demographics (sex, age, influenza subtype), laboratory findings [complete blood count (WBC, NEU, LYM, MONO), complement assays (C3, C4), lymphocyte subsets (CD3+ CD4+ T cell, CD3+ T cell, CD3+ CD8+ T cell, CD19+ B cells, NK cells), and cytokines (IL-6, IL-10, IL-2, TNF-α, IL-4)], and clinical course metrics (time from fever onset to sampling, prior glucocorticoid/IVIG use, and ≥2 pre-diagnostic convulsive episodes).

Regarding the clinical availability of laboratory tests: All immunological parameters, including the cytokine panel (IL-2, IL-4, IL-6, IL-10, TNF-α), lymphocyte subsets, and complement assays, were part of the routine clinical laboratory testing menu at Wuhan Children’s Hospital during 2020–2025, clinically indicated for pediatric patients with febrile illness, suspected sepsis, or neurological complications. All tests were performed on blood samples collected at admission (median time from fever onset to sampling: 24 h, IQR: 20–48 h). Prior use of immunomodulatory agents (e.g., IVIG, glucocorticoids) before sampling was recorded as a binary variable, and both this variable and the time-from-fever-onset-to-sampling window were included as covariates in multivariable models to adjust for potential confounding.

### 2.3. Statistical Analysis

#### 2.3.1. Data Distribution and Group Comparisons

Normality of continuous variables was assessed using the Shapiro–Wilk test. Normally distributed data were expressed as mean ± standard deviation (SD) and compared using Student’s *t*-test. Non-normally distributed data were presented as median (interquartile range [IQR]) and analyzed using the Mann–Whitney U test. For multivariable logistic regression, continuous variables with skewed distributions (e.g., IL-6, NEU, C3) were analyzed in their original units. Given the potential for skewed distributions to influence model estimates, we additionally performed sensitivity analyses using log-transformed values and IQR-standardized values, which yielded consistent results (Supplementary Table S1). This approach ensured that the handling of skewed variables was consistent between descriptive and regression analyses. Categorical variables were reported as frequencies (*n*, %) and compared using the chi-square (χ^2^) test or Fisher’s exact test, as appropriate. Violin plots were generated to visualize the distributional differences in key variables between the IAE and FS groups.

#### 2.3.2. Collinearity Assessment and Variable Selection

To mitigate multicollinearity among candidate predictors, variance inflation factors (VIFs) were calculated for all variables. WBC and NEU exhibited high collinearity (VIF > 9); therefore, WBC was excluded from subsequent regression models to avoid multicollinearity, and NEU was retained as the representative myeloid marker. Variables demonstrating statistical significance in univariate analyses (*p* < 0.05) were entered into multivariable logistic regression models to identify independent risk factors for IAE.

#### 2.3.3. Sequential Logistic Regression Models

Four sequential multivariable logistic regression models were constructed to control for potential confounders: Model 1 (crude model, without adjustment); Model 2 (adjusted for demographic characteristics: age, sex, and influenza subtype); Model 3 (adjusted for clinical management and severity: time from fever onset to sampling, prior use of glucocorticoids, prior use of intravenous immunoglobulin [IVIG], and the presence of ≥2 pre-diagnostic convulsive episodes); and Model 4 (fully adjusted model incorporating all variables from Models 2 and 3). Given the limited number of IAE events (n = 55), Model 4 was considered exploratory, and the primary interpretation was based on Models 1–3. To further assess the stability of Model 4 estimates, we performed bootstrap internal validation (1000 resamples) and Firth’s penalized logistic regression as sensitivity analyses, which yielded consistent results (Supplementary Table S1). Forest plots visualizing the regression estimates were constructed using GraphPad Prism (version 10.1.2; GraphPad Software, Boston, MA, USA).

#### 2.3.4. Sensitivity Analysis for C3 Floor Effects

To address potential floor effects in complement C3 measurements, we performed a sensitivity analysis in which C3 values below the 5th percentile (0.70 g/L) were Winsorized to this threshold. The multivariable model was then refitted, adjusting for the same covariates as the primary model (age, sex, neutrophil count, CD3+ CD4+ T cells, CD19+ B cells, and IL-6). The results of this sensitivity analysis are presented in Supplementary Table S2.

#### 2.3.5. Software and Significance Threshold

All statistical analyses were performed using R software (version 4.4.3) and SPSS Statistics (version 25.0). Statistical significance was defined as a two-tailed *p* < 0.05 throughout the study.

## 3. Results

### 3.1. Comparison of General Situation and Laboratory Test Results

A total of 623 pediatric influenza cases with fever and convulsion symptoms were included, among which 394 were male (63.2%) and 229 were female (36.8%). The IAE group consisted of 55 cases, including 28 males and 27 females. The IAE group had a significantly higher proportion of children aged >60 months compared to the FS group (41.8% vs. 25.5%, *p* = 0.018). Compared to the FS group, the IAE group exhibited significantly lower levels of C3, C4, and CD3+ CD4+ T cells, and significantly higher levels of NEU, IL-6, and IL-10 (all *p* < 0.05, Table 1).

### 3.2. Distribution of Significant Variables Between IAE and FS Groups

**Figure 2:**
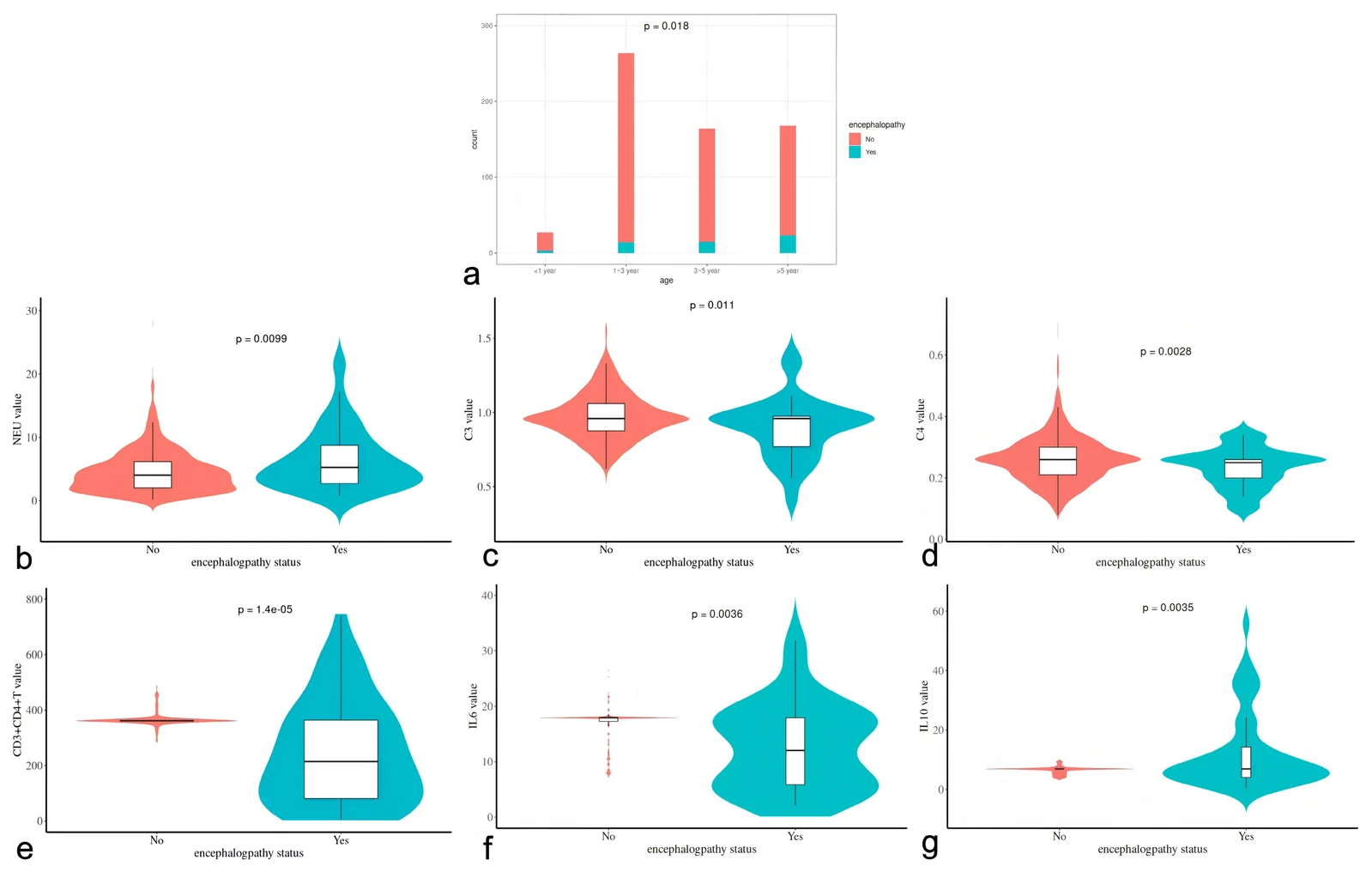
Violin plots of significantly different variables between the two groups. (**a**) Proportions of categorical variables (stacked bar chart). (**b**–**d**) Violin/box plots of complement components C3, C4, and IL-6 levels by encephalopathy status. (**e**–**g**) Violin/box plots of lymphocyte subsets and IL-10 levels (CD3+ CD4+ T-cell count, NK-cell count, and IL-10) by encephalopathy status. Data are shown as violin plots with embedded box plots (median and interquartile range); *p* values were obtained by the Mann–Whitney U test (or chi-square test for panel a). IAE, influenza-associated encephalopathy; C3/C4, complement components 3/4; CD3+ CD4+, cluster of differentiation 3/CD4+ T cells; NK, natural killer cells; IL, interleukin.

The violin plots (Figure 2) illustrated significant distributional disparities across seven key indicators. Compared to the FS group, the IAE cohort exhibited an older age distribution, right-skewed elevations in NEU, IL-6, and IL-10 (indicating hyperinflammation), and significant downward shifts in C3, C4, and CD3+ CD4+ T cells (reflecting immunosuppression). These findings visually confirm the “hyperinflammation and low immunity” phenotype in IAE.

### 3.3. Univariate Analysis of Risk Factors

Univariate logistic regression analysis indicated that older age (OR = 1.011, 95% CI: 1.003–1.018, *p* = 0.008) and male sex (OR = 1.747, 95% CI: 1.002–3.046, *p* = 0.049) were associated with an increased risk of IAE. Additionally, elevated levels of WBC, NEU, IL-6, IL-10, and CD19+ B cells were significantly associated with an increased risk of IAE, while decreased levels of CD3+ CD4+ T cells, C3, and C4 were also significantly associated with the outcome (Table 2).

### 3.4. Multicollinearity Diagnosis and Variable Selection

**Figure 3:**
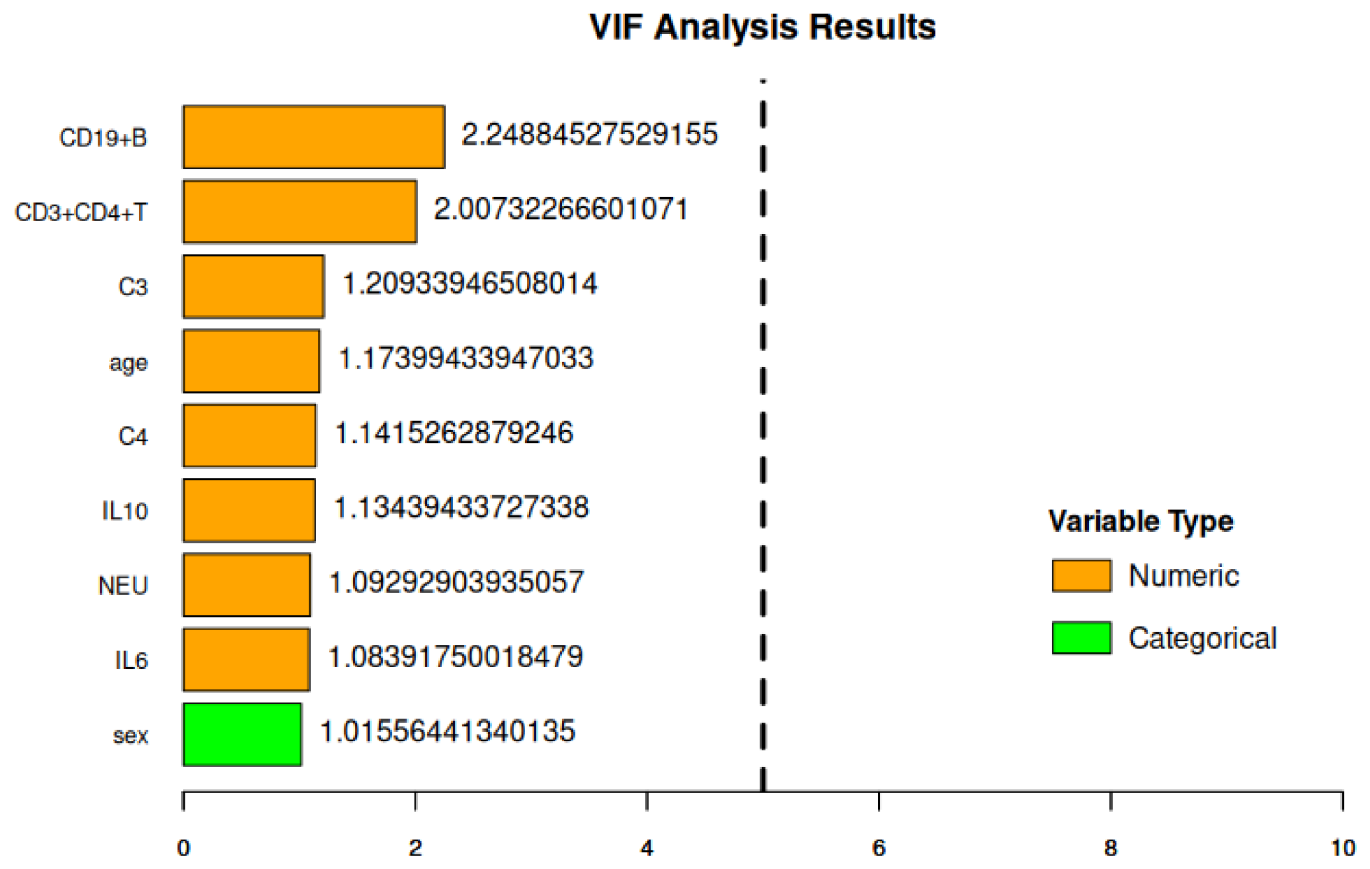
Ranking of variables based on Variance Inflation Factor (VIF) values. All variables showed VIF values < 5 (well below the conventional threshold of 10), indicating no substantial multicollinearity. CD19+ B, CD19+ B-cell count; CD3+ CD4+ T, CD3+ CD4+ T-cell count; NEU, neutrophil count; age, age; sex, sex (categorical).

To avoid the impact of multicollinearity on the subsequent multivariate model, a collinearity diagnosis was performed on the indicators that were statistically significant in the univariate analysis. The VIF was calculated to assess the collinearity among variables, and the variables were ranked according to their VIF values (Figure 3). Generally, a VIF > 5 indicates moderate collinearity, and a VIF > 10 indicates severe collinearity. The VIF analysis revealed that WBC (VIF = 9.544) and NEU (VIF = 9.454) exhibited high collinearity. To avoid multicollinearity in the multivariable model, WBC was excluded, and NEU was retained as the representative myeloid marker. All other candidate variables had a VIF < 5, indicating no severe multicollinearity among the remaining variables.

### 3.5. Multivariable Logistic Regression Analysis

To identify independent risk factors for IAE, we constructed four sequentially adjusted multivariable logistic regression models (Table 3). In the fully adjusted Model 4, elevated neutrophil (NEU) counts remained significantly associated with an increased risk of IAE (adjusted OR = 1.131, 95% CI: 1.027–1.244, *p* = 0.012). Regarding lymphocyte subsets, decreased counts of CD3+ CD4+ T cells (adjusted OR = 0.983, 95% CI: 0.979–0.988, *p* < 0.001) and elevated CD19+ B cells (adjusted OR = 1.007, 95% CI: 1.003–1.010, *p* < 0.001) were both significantly associated with IAE. Elevated IL-6 levels maintained their significant association with IAE risk (adjusted OR = 1.002, 95% CI: 1.000–1.003, *p* = 0.037). In the primary analysis, lower C3 levels were significantly associated with IAE (adjusted OR = 0.05, 95% CI: <0.001–0.138, *p* = 0.002). A sensitivity analysis using 5th-percentile Winsorization of C3 yielded a similar estimate (adjusted OR = 0.019, 95% CI: 0.002–0.226), with the direction and significance of other covariates remaining unchanged (Supplementary Table S2). These findings indicate that the association between low C3 and IAE was robust and not solely attributable to floor effects in C3 measurement.

**Figure 4:**
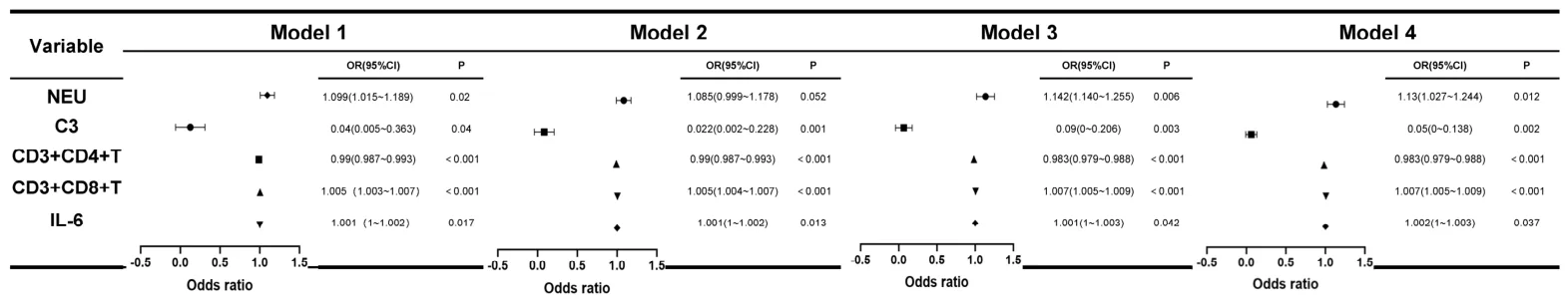
Forest plot of multivariate-adjusted associations between immunological markers and influenza−associated encephalopathy: A comparison of four nested regression models. Different symbols (circle, diamond, square, triangle, inverted triangle) are only used to distinguish the distribution of each indicator and carry no other special meanings. **Model specifications:** Model 1: unadjusted; Model 2: adjusted for sex, age, and influenza type; Model 3: adjusted for time from fever onset to sampling, pre-sampling use of corticosteroids and IVIG, and recurrent convulsions; Model 4: adjusted for all aforementioned variables.

The consistency of these findings across the four nested models was visualized using forest plots (Figure 4), supporting the overall robustness of the observed associations.

## 4. Discussion

This study identifies a distinct immunological landscape of pediatric influenza-associated encephalopathy (IAE) through comprehensive immunophenotyping of 623 pediatric influenza patients with febrile seizures. Our findings characterize IAE as a “hyperinflammation-with-immunosuppression” phenotype. We propose a “two-hit” model of innate-adaptive immune dysregulation as a hypothesis-generating framework. While systemic complement dysregulation and cytokine elevation were prominent features, CD3+ CD4+ T cell depletion remained significantly associated with IAE across all sequential adjustment models, suggesting that adaptive immune suppression may be a central component of IAE susceptibility. However, causal relationships cannot be established from this observational dataset.

The first hit of this proposed model involves innate immune hyperactivation. We observed significantly elevated IL-6 levels and lower C3 levels in IAE patients. Experimental evidence supports the pathogenic relevance of complement dysregulation: C3-deficient mice exhibit exacerbated pulmonary inflammation and delayed viral clearance during IAV infection [5], and the globular domain of C1q directly inhibits IAV entry and modulates inflammatory responses [6]. Analogously, complement activation has been extensively documented in severe COVID-19, where C3 upregulation and downstream C5b-9 assembly contribute to systemic and neuroinflammatory injury, suggesting a shared mechanistic theme across severe viral infections [13]. These observations raise the hypothesis that complement dysregulation may contribute to systemic hyperinflammation in IAE. However, whether lower C3 levels reflect complement consumption, impaired synthesis, or an epiphenomenon of systemic inflammation cannot be determined from our data. Uncontrolled systemic hyperinflammation may compromise blood–brain barrier (BBB) integrity, permitting peripheral mediators to access the CNS, but this sequence requires direct experimental validation. At the molecular level, RANBP2/NUP358 has been implicated in regulating IAV-induced hyperinflammation [4]; however, how this nuclear pore pathway scales into systemic C3-IL-6 phenotypes warrants further investigation.

The second hit of this proposed model is the depletion of the CD4+ T cell compartment. CD3+ CD4+ T cell depletion remained significantly associated with IAE across all four sequential adjustment models, including Model 3, which accounted for sampling timing and prior immunomodulatory therapy. This consistency across models, further supported by sensitivity analyses using alternative transformations of skewed variables (Supplementary Table S1), suggests that cellular immune dysfunction may be less susceptible to transient clinical interventions than humoral markers. However, formal comparison of predictive performance (e.g., AUC, reclassification metrics) would be needed to establish whether CD4+ T cell count is superior to other markers for risk stratification. This aligns with emerging evidence in H1N1-associated acute necrotizing encephalopathy, where pediatric patients exhibit a striking reduction in peripheral CD4+ T cell counts accompanied by heightened expression of exhaustion markers (Eomes, CD69, PD-1) and concordant surges in IL-6 and IL-8 within both cerebrospinal fluid and plasma [7]. Earlier work further documented upregulated CTLA-4 expression on CD4+ T cells in children with IAV encephalopathy relative to those without neurological complications [9]. Mechanistically, CD4+ T cells are indispensable for coordinating protective anti-influenza immunity—providing helper signals for B cell antibody production, licensing CD8+ T cell cytotoxicity, and sustaining inflammatory resolution [10]; their depletion or functional paralysis may therefore impair viral clearance while failing to restrain the cytokine storm.

Elevated IL-6 levels corroborated prior reports linking IL-6 to IAE severity and mortality [8,11]. IL-6 promotes cell survival via STAT3/NF-κB signaling and, together with other pro-inflammatory cytokines, has been reported to disrupt BBB tight junctions (Occludin, Claudin-5, ZO-1) in experimental models [3]. Consistent with the broader literature on viral encephalopathies, the predominant IAE pathology across larger clinical series is host-driven, cytokine-mediated injury without direct CNS viral replication [3]. While acute necrotizing encephalopathy can also occur with non-influenza pathogens such as HHV-6 [14], the immunological signature we identified—marked by CD4+ T cell exhaustion superimposed on innate immune hyperactivation—appears to be a common endpoint of severe viral CNS injury. Intracranially, IAV-triggered glial activation releases neurotoxins and alters lipid metabolism, compounding neuronal injury [3].

Regarding cellular immunity, reductions in CD3+ CD4+ T cells reflected suppressed host defenses, whereas elevated CD19+ B cells emerged as an independent risk factor in our cohort. Recent work in critical pediatric influenza has similarly established CD19+ B cells as an independent risk factor [12]. In severe viral infections, aberrant B-cell activation has been reported to stem from CD4+ T cell dysfunction [15]. We hypothesize a “T-B immune dysregulation axis” in IAE, in which impaired regulatory capacity of CD4+ T cells fails to constrain B-cell activity, potentially driving aberrant expansion. Persistent antigen exposure may drive CD4+ T exhaustion via the TNF-NF-κB/PD-1 axis, suppressing antiviral immunity and exacerbating immunopathology [15]; however, this mechanism requires direct experimental validation in IAE.

This study has several limitations. First, reliance solely on peripheral blood precludes direct confirmation of whether immune cells infiltrate the CNS via the damaged BBB. Second, prior administration of corticosteroids or IVIG before sampling may have interfered with immunological metrics, although our sequential adjustment models (particularly Model 3) were designed to mitigate this confounding. Third, the retrospective, single-center design limits causal inference. Fourth, the limited number of IAE events (*n* = 55) restricted statistical power and increased the risk of model overfitting, particularly in the fully adjusted Model 4; therefore, results from Model 4 should be interpreted as exploratory. Future longitudinal studies with paired peripheral blood and cerebrospinal fluid sampling, as well as murine IAE models, are warranted to dissect the temporal dynamics of this immune dysregulation.

The dual nature of the observed immunophenotype—hyperinflammation and immunosuppression—raises the possibility that immunomodulatory strategies, if considered in future studies, would need to balance anti-inflammatory and immune-restorative approaches. For example, IL-6-targeted therapies, complement pathway modulators, or immune-stratified treatment escalation have been proposed in other severe viral inflammatory conditions [16,17,18,19]. However, the present observational data cannot establish whether modulating IL-6, complement, or T cell pathways would improve clinical outcomes in IAE. Interventional studies are required before any therapeutic recommendations can be made.

## 5. Conclusions

In summary, this study identifies a distinct “hyperinflammation-with-immunosuppression” immunophenotype in pediatric IAE, characterized by lower C3 levels, IL-6-dominated inflammation, and profound CD3+ CD4+ T cell depletion. We propose a “two-hit” model-innate immune dysregulation followed by adaptive immune paralysis-as a hypothesis-generating framework. CD4+ T cell metrics remained significantly associated with IAE after adjustment for clinical confounders, supporting their potential utility in early risk stratification. Given the observational design and limited number of IAE events, these findings should be considered hypothesis-generating; causal relationships and therapeutic implications warrant investigation in prospective cohorts and mechanistic studies.

## Figures and Tables

**Table 1:** Comparison of laboratory tests between the IAE group and the non-IAE group.

Characteristics	FS Group (n = 568)	IAE Group (n = 55)	*p* Value
**sex (female)**	202 (35.6)	27 (49.1)	0.066
**Age**			**0.018**
**1~12 m**	24 (4.2)	3 (5.5)	
**13~36 m**	250 (44)	14 (25.5)	
**37~60 m**	149 (26.2)	15 (27.3)	
**>** **60 m**	145 (25.5)	23 (41.8)	
**Flu (flu-a)**	451 (79.4)	47 (85.5)	0.285
**WBC (10^9^/L)**	6.32 [4.73, 8.52]	7.35 [4.64, 11.62]	0.133
**NEU (10^9^/L)**	4.03 [2.03, 6.20]	5.26 [2.74, 8.75]	**0.009**
**LYM (10^9^/L)**	1.44 [0.88, 2.29]	1.3 [0.63, 2.02]	0.13
**MONO (10^9^/L)**	0.53 [0.37, 0.74]	0.45 [0.21, 0.92]	0.116
**C3 (g/L)**	0.96 [0.88, 1.06]	0.96 [0.77, 0.98]	**0.011**
**C4 (g/L)**	0.26 [0.21, 0.30]	0.25 [0.20, 0.26]	**0.02**
**CD3+ CD4+ T (count/μL)**	362 [352, 412.25]	225 [82, 374]	**<0.001**
**CD3+ T (count/μL)**	733 [700, 766]	631 [334, 986]	0.28
**NK (count/μL)**	129.5 [124.5, 134]	107 [54, 198]	0.55
**CD19+ B (count/μL)**	323.50 [285, 323.5]	362 [219, 662.5]	0.275
**CD3+ CD8+ T (count/μL)**	240 [234.5, 280]	245 [112, 385]	0.991
**IL-6 (pg/mL)**	17.94 [15.1, 20.5]	57.94 [49.71, 152.5]	**0.02**
**IL-10 (pg/mL)**	6.92 [5.91, 7.49]	8.85 [5.26, 34.14]	**0.018**
**IL-2 (pg/mL)**	2.2 [1.83, 2.41]	2.71 [1.79, 3.35]	0.23
**TNF-α (pg/mL)**	2.64 [2.48, 3]	2.53 [1.88, 3.39]	0.051
**IL-4 (pg/mL)**	2.03 [1.81, 2.21]	2.05 [1.63, 2.9]	0.292

**Abbreviations:** FS, febrile seizure; IAE, influenza-associated encephalopathy; WBC, white blood cell count; NEU, neutrophil count; LYM, lymphocyte count; MONO, monocyte count; C3, complement component 3; C4, complement component 4; CD, cluster of differentiation; NK, natural killer cell; IL, interleukin; TNF-α, tumor necrosis factor-alpha.

**Table 2:** Univariate Logistic Regression Analysis of IAE.

Characteristics	*β*	OR	95% CI	*p*	Tolerance	VIF
**sex**	0.558	1.747	1.002~3.046	0.049	0.985	1.016
**age**	0.011	1.011	1.003~1.018	0.008	0.799	1.251
**WBC**	0.089	1.093	1.032~1.158	0.002	0.105	9.544
**NEU**	0.106	1.112	1.045~1.183	0.001	0.106	9.454
**C3**	−2.98	0.051	0.08~0.31	0.001	0.824	1.213
**C4**	−5.484	0.004	0~0.245	0.008	0.869	1.151
**CD3+ CD4+ T**	−0.006	0.994	0.992~0.996	<0.001	0.493	2.029
**CD19+ B**	0.001	1.001	1~1.001	0.013	0.439	2.279
**IL-6**	0.001	1.001	1~1.002	0.004	0.923	1.084
**IL-10**	0.008	1.008	1.004~1.011	<0.001	0.869	1.151

Data are presented as regression coefficients (*β*), odds ratios (OR) with 95% confidence intervals (CI), *p* values, and collinearity diagnostics (tolerance and variance inflation factor [VIF]). C3, complement component 3; C4, complement component 4; CD3+ CD4+ T, CD3+ CD4+ T-cell count; CD19+ B, CD19+ B-cell count; IL, interleukin; WBC, white blood cell count; NEU, neutrophil count. Statistically significant predictors are shown in bold (*p* < 0.05).

**Table 3:** Associations between immunological markers and influenza-associated encephalopathy: results from four sequentially adjusted regression models.

Characteristics		Model 1			Model 2			Model 3			Model 4	
	β	OR (95% CI)	*p*	β	OR (95% CI)	*p*	β	OR (95% CI)	*p*	β	OR (95% CI)	*p*
NEU	0.094	1.099 (1.015~1.189)	0.02	0.082	1.085 (0.999~1.178)	0.052	1.133	1.142 (1.140~1.255)	0.006	0.122	1.13 (1.027~1.244)	0.012
C3	−3.207	0.04 (0.005~0.363)	0.04	−3.799	0.022 (0.002~0.228)	0.001	−4.667	0.09 (<0.001~0.206)	0.003	−5.327	0.05 (<0.001~0.138)	0.002
CD3+ CD4+ T	−0.01	0.99 (0.987~0.993)	<0.001	−0.01	0.99 (0.987~0.993)	<0.001	−0.017	0.983 (0.979~0.988)	<0.001	−0.017	0.983 (0.979~0.988)	<0.001
CD19+ B	0.005	1.005 (1.003~1.007)	<0.001	0.005	1.005 (1.004~1.007)	<0.001	0.007	1.007 (1.005~1.009)	<0.001	0.007	1.007 (1.005~1.009)	<0.001
IL-6	0.001	1.001 (1~1.002)	0.017	0.001	1.001 (1~1.002)	0.013	0.001	1.001 (1~1.003)	0.042	0.002	1.002 (1~1.003)	0.037

**Model specifications:** Model 1: unadjusted; Model 2: adjusted for sex, age, and influenza type; Model 3: adjusted for time from fever onset to sampling, pre-sampling use of corticosteroids and IVIG, and recurrent convulsions; Model 4: adjusted for all aforementioned variables.

## Data Availability

The datasets used and/or analyzed during the current study are available from the corresponding author (bloveriver@163.com) upon reasonable request.

## Supplementary Materials

The following supporting information can be downloaded at: https://www.mdpi.com/article/10.3390/diagnostics16203290/s1, Table S1: Sensitivity analyses for skewed continuous variables using alternative transformations in multivariable logistic regression models for IAE. Table S2: Sensitivity analyses for complement C3 in the prediction of pediatric influenza-associated encephalopathy (IAE).

## Abbreviations

The following abbreviations are used in this manuscript:

IAV	Influenza A virus
IAE	influenza-associated encephalopathy
FS	simple febrile seizure
IA-ANE	influenza-associated acute necrotizing encephalopathy
NSAIDs	Nonsteroidal Anti-inflammatory Drugs
GCS	Glasgow Coma Scale
WBC	white blood cells
NEU	neutrophils
LYM	lymphocytes
MONO	monocytes
SD	standard deviation
VIF	variance inflation factor
BBB	blood–brain barrier
IVIG	intravenous immunoglobulin
