# Supplementary material for: Convergent Immunopathology in Influenza-Associated Encephalopathy: CD3+ CD4+ T Cell Depletion, IL-6-Mediated Inflammation, and Complement C3 Dysregulation
Source: Diagnostics (Basel). 2026 Oct 10;16(20):3290. doi: 10.3390/diagnostics16203290 (PMC13703285; doi:10.3390/diagnostics16203290)
Supplement: Supplementary file 1 [file diagnostics-16-03290-s001.zip › diagnostics-4506859-supplementary.pdf]

Supplementary Table S1. Sensitivity analyses for skewed continuous variables using alternative transformations in multivariable logistic regression models for IAE

Multivariable logistic regression models were adjusted for age, sex, influenza subtype, time from fever onset to sampling, prior use of glucocorticoids, prior use of intravenous immunoglobulin, and the presence of ≥2 pre-diagnostic convulsive episodes (Model 4). Skewed continuous variables (IL-6, NEU, C3) were analyzed in their original units, after natural log-transformation, and after standardization by the interquartile range (IQR). CD3+CD4+ T cells and CD19+ B cells were included in all models without transformation. ORs for log-transformed variables represent the change in odds per one-unit increase in the natural log of the variable; ORs for IQR-standardized variables represent the change in odds per one-IQR increase in the variable. All p values are two-sided.

Variable	Primary analysis (original units)	Log-transformed	IQR-standardized
	OR (95% CI), p	OR (95% CI), p	OR (95% CI), p
**IL-6**	1.002(1-1.003),0.037	3.158(1.465-6.808),0.003	1.011(1.001-1.02),0.037
**NEU**	1.13(1.027-1.244),0.012	5.299(1.414-19.865),0.013	1.673(1.104-2.536),0.015
**C3**	0.05(＜0.001-0.138),0.002	＜0.001(＜0.001-0.025),0.002	0.35(0.191-0.64),0.001
**CD3+CD4+T**	0.983(0.979-0.988),＜0.001	0.982(0.978-0.987),＜0.001	0.983(0.978-0.987),＜0.001
**CD19+B**	1.007(1.005-1.009),＜0.001	1.007(1.005-1.009),＜0.001	1.007(1.005-1.01),＜0.001

Abbreviations: IAE, influenza-associated encephalopathy; OR, odds ratio; CI, confidence interval; IL-6, interleukin-6; NEU, neutrophil count; C3, complement component 3; CD, cluster of differentiation; IQR, interquartile range.

Supplementary Table S2. Sensitivity analyses for complement C3 in the prediction of pediatric influenza-associated encephalopathy (IAE).

Multivariable logistic regression models were adjusted for age, sex, neutrophil count, CD3+CD4+T cells, CD19+ B cells, and IL-6.

Variable	Model 1: Primary Cohort (Original C3)	Model 2: Winsorized C3 (C3 < 0.70 → 0.70 g/L)
**Complement C3**	**0.015 (0.002–0.146)** ***	**0.019 (0.002–0.226)** *
Age (per month)	1.019 (1.008–1.030) **	1.018 (1.008–1.029) **
Sex (Male vs. Female)	2.189 (1.091–4.395) *	2.278 (1.142–4.547) *
Neutrophils (NEU)	1.083 (0.998–1.175)	1.079 (0.995–1.170)
CD3+CD4+ T cells	0.990 (0.987–0.993) ***	0.990 (0.987–0.993) ***

Variable	Model 1: Primary Cohort (Original C3)	Model 2: Winsorized C3 (C3 < 0.70 → 0.70 g/L)
CD19+ B cells	1.005 (1.004–1.007) ***	1.005 (1.004–1.007) ***
IL-6 (per pg/mL)	1.001 (1.000–1.002) *	1.001 (1.000–1.002) *
**Observations (n)**	**623**	**623**
**IAE Cases (n)**	**55**	**55**

Data are presented as Odds Ratio (95% Confidence Interval). *P<0.05, **P<0.01, ***P<0.001. Model 2 addresses potential floor effects by recoding extreme low C3 values to the 5th percentile threshold (0.70 g/L).*
